# Development of a Green Capillary Electrophoresis Method for Determining and Quality Control of Dapagliflozin: An Oral Hypoglycemic Agent

**DOI:** 10.1002/elps.70042

**Published:** 2025-09-28

**Authors:** Lívia Maronesi Bueno, Manoelly Oliveira Rocha, Amanda Mohr, Andreia Neves Fernandes, Martin Steppe

**Affiliations:** ^1^ Laboratory of Pharmaceutical Quality Control Federal University of Rio Grande Do Sul Porto Alegre Rio Grande do Sul Brazil; ^2^ Chemistry Institute Federal University of Rio Grande Do Sul Porto Alegre Rio Grande do Sul Brazil

**Keywords:** capillary electrophoresis | dapagliflozin | drug analyses | green method

## Abstract

A green method by capillary electrophoresis (CE) is described for the first time for the determination of dapagliflozin (DAPA), an oral hypoglycemic drug approved for the treatment of Type 2 diabetes mellitus. The effects of different analytical conditions were evaluated, including the concentration and pH of the background electrolyte (BGE), sample injection time, applied voltage, as well as capillary temperature. The method was validated by establishing the linearity, intra‐ and interday precisions (relative standard deviation, RSD%), accuracy, and robustness. The analytical procedure was linear in the range of 50–175 µg mL^−1^ (*R* ^2^ > 0.999), with the limit of detection (LOD) and limit of quantitation (LOQ) of 6.2 and 18.8 µg mL^−1^, respectively. Precision had an intraday RSD of 2.55% and an interday RSD of 2.52%. The average recovery rates for the pharmaceutical samples ranged from 101.22% to 104.63%, with an RSD of 0.88%. Additionally, the CE method was compared to a high‐performance liquid chromatography (HPLC) method for quantifying DAPA, and their green profiles were assessed by the Analytical Greenness Metric (AGREE), confirming the eco‐friendliness of the CE technique. The methodology is suitable for determining DAPA in tablets; CE provides a greener alternative due to low‐cost analysis using fewer organic solvents and minimizing waste generation.

**Abbreviations**
AGREE: Analytical Greenness Metric.
ANOVA: analysis of variance
CE: capillary electrophoresis
DAPA: dapagliflozin
GAC: green analytical chemistry
HPLC: high‐performance liquid chromatography
ICH
: International Council for Harmonization
IS: internal standard
LOD: limit of detection
LOQ: limit of quantification
RSD: relative standard deviation
USP: United States Pharmacopeia

## 1 Introduction

Diabetes mellitus is a major global health concern, affecting millions of individuals worldwide [1]. According to the World Health Organization (WHO), the number of people living with diabetes has increased significantly in recent decades, reaching approximately 830 million, with Type 2 diabetes being the most prevalent form. Pharmacological intervention is often required to regulate blood glucose levels and prevent diabetes‐related complications. However, more than half of those affected do not receive adequate pharmacological treatment [2].

Currently, several classes of hypoglycemic agents are available, with different mechanisms of action and dosages. Dapagliflozin (DAPA) is the first approved sodium–glucose cotransporter‐2 (SGLT2) inhibitor, a modern and promising class for treating Type 2 diabetes. It helps to improve glycemic control by reducing the renal reabsorption of glucose, leading to glucose excretion and weight loss. Additionally, the drug has been shown to improve cardiovascular conditions [3, 4].

**FIGURE 1:**
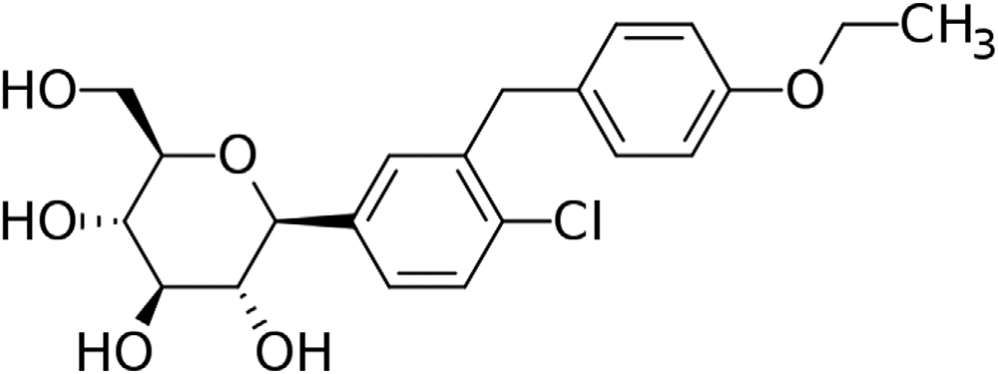
Chemical structure of DAPA.

Effective analytical methods for determining DAPA (Figure 1) are vital to ensuring that the drug meets the required standards for purity, concentration, and stability, ultimately guaranteeing the safe release of the medication to the market. The literature survey shows several high‐performance liquid chromatography (HPLC) methods for the determination of DAPA in biological samples [5, 6, 7] as well as in pharmaceutical formulations [8, 9, 10, 11, 12]. In addition, a high‐performance thin‐layer chromatography (HPTLC) method has been reported for the simultaneous determination of metformin hydrochloride, vildagliptin, and DAPA propanediol monohydrate, which was further evaluated using green analytical metrics to assess its environmental impact [13]. A voltammetric method has also been reported for the investigation of DAPA [14], and only one capillary electrophoresis (CE) was found, which is proposed for the simultaneous determination of metformin hydrochloride, saxagliptin hydrochloride, and DAPA [15].

CE is a technique whose main advantage is reducing samples and solvents, contributing to a more sustainable and less expensive analysis. The methods require minimal sample and solvent volumes due to the small internal volume of the capillary column, leading to a considerable reduction in waste generation. Additionally, the background electrolyte (BGE) is primarily aqueous, leading to a significant decrease in organic solvent use. Furthermore, CE benefits from lower energy consumption relative to techniques like HPLC, contributing to a reduced environmental impact. However, it is important to recognize that CE presents certain challenges, particularly regarding reproducibility. Slight variations in conditions can affect the consistency of results, impacting their reliability for quantitative analysis in certain applications. As highlighted in the literature, achieving reproducibility requires careful method optimization and proper conditioning of the capillary [16, 17, 18, 19].

All these mentioned inherent characteristics and advantages of CE are closely aligned with green analytical chemistry (GAC) principles. In general, GAC aims to promote more environmentally friendly practices in laboratories. The key goals in greening analytical methodologies include reducing chemicals, minimizing energy consumption, properly managing and reducing analytical waste, and increasing the operator's safety [20]. These sustainable practices are increasingly adopted in pharmaceutical analysis, where environmental impact and resource utilization are critical considerations. Although some of the reported HPLC methods for determining DAPA explore GAC concepts, the CE technique stands out regarding sustainability [18].

The present study aimed to develop and validate an analytical method based on CE for quantifying DAPA in pharmaceutical dosage form, seeking to provide a robust, efficient, and environmentally sustainable alternative. Additionally, the proposed CE method was compared with a previously established HPLC method in terms of analysis time, solvent consumption, waste generation, and separation efficiency. The method of greenness was also evaluated by applying a green metric.

## 2 Materials and Methods

### 2.1 Instruments and Analytical Conditions

CE experiments were performed using an Agilent 3DCE system (Agilent Technologies, Germany) equipped with an autosampler, a photodiode array detector, and a temperature controller. Data acquisition and analysis were carried out using CE ChemStation software.

All experiments were conducted in a fused silica capillary with an internal diameter of 50 µm and a total length of 48.5 cm (40 cm effective length). The new capillaries were initially activated by flushing with 1.0 M NaOH for 15 min, followed by rinsing with purified water for 10 min. Prior to each use, the capillaries were conditioned by sequential rinsing with 0.1 M NaOH for 5 min, purified water for 4 min, and the BGE for 5 min. Between successive measurements, the capillaries were reconditioned using 0.1 M NaOH for 3 min, followed by purified water for 1.5 min, and the BGE for 3 min. After each day of use, the capillary was rinsed with distilled water and then dried by applying pressure using an empty vial to remove any residual bubbles or liquid. This procedure prevents blockage and drying inside the capillary during overnight storage or on days when the instrument is not in use, thus ensuring consistent performance. The BGE consisted of a 40 mM sodium tetraborate solution, adjusted to pH 10.5 with NaOH.

The electrophoretic conditions were optimized by applying a voltage of 14 kV (current approximately 52 µA), maintaining a temperature of 25°C, performing hydrodynamic injection at 50 mBar for 5 s, and setting the detection wavelength to 230 nm. At the beginning of each analysis day and between each injection, the capillary was preconditioned to guarantee the reproduction of results.

### 2.2 Chemicals and Reagents

DAPA (98.4%) reference substance was purchased from Ontario Chemical (Canada). DAPA 10 mg tablets (Forxiga, AstraZeneca) were obtained from the local market. Sodium phosphate monobasic, boric acid, TRIS–HCl buffer, sodium tetraborate, sodium hydroxide, hydrochloric acid, and acetonitrile were obtained from Sigma‐Aldrich (Germany). Ultrapure water (Direct‐Q3UV, France) was used to prepare solutions. Ranitidine (99.9%), used as an internal standard (IS), was obtained from the United States Pharmacopeia (USP) (Rockville, MD, USA).

### 2.3 Stock Solution Preparations

The DAPA stock standard solution was prepared by accurately weighing the reference substance and dissolving it in acetonitrile to obtain a final concentration of 1000 µg mL^−1^. The IS solution was similarly prepared by dissolving an appropriate amount of ranitidine reference standard in ultrapure water at a concentration of 1000 µg mL^−1^. For the preparation of the sample solution, 20 DAPA tablets were accurately weighed, finely powdered, and homogenized. A portion of the powdered material, equivalent to 10 mg of DAPA, was transferred to a 10 mL volumetric flask. Extraction was performed using acetonitrile as the solvent under sonication for 30 min to ensure complete release of the active pharmaceutical ingredient from the tablet matrix. The resulting solution was then brought to volume with the same solvent.

The placebo solution was prepared by weighing all the excipients (microcrystalline cellulose, lactose, crospovidone, silicon dioxide, magnesium stearate, film‐coating polyvinyl alcohol, titanium dioxide, macrogol 3350, talc, and iron oxide yellow) of the dosage form in their usual concentration according to the Handbook of Pharmaceutical Excipients [21] and prepared in the same way as the sample solution.

All the working solutions, prior to measurements, were diluted in the BGE until the final concentration was 75 µg mL^−1^, and filtered through a 0.45 µm membrane filter (Millipore Corp.). The solutions used in preconditioning (0.1 M NaOH, water, and BGE) were filtered similarly.

### 2.4 Method Validation

The developed method was validated according to the USP requirements and International Council for Harmonization (ICH Q2(R1)) guidelines, where the parameters of selectivity, linearity, limits of detection (LOD) and quantification (LOQ), precision, accuracy, and robustness were evaluated [22, 23]. For the validation studies, the DAPA stock solutions were properly diluted with the optimized BGE to obtain the working concentration required for each assay. To ensure accuracy, consistency, and reproducibility of the quantification process, the IS was added at a fixed concentration to all standard and sample solutions prior to injection. This procedure was systematically applied across all validation steps, in accordance with ICH Q2(R1) guidelines for quantitative analytical methods.

#### 2.4.1 Selectivity

Selectivity was evaluated by assessing potential interference from excipients and degradation products formed under acidic, alkaline, oxidative, photolytic (UVA/UVC), and thermal stress conditions [22, 23]. For chemical degradation, stock solution aliquots were mixed with 1 M HCl, 1 M NaOH, or 3% H_2_O_2_. Photostability was tested under UVA (352 nm) and UVC (254 nm) light (200 Wh m^−2^), and thermal stress was applied at 60°C using tightly sealed vials to prevent solvent evaporation during storage. Samples were collected hourly over 5 h, adjusted to 75 µg mL^−1^, neutralized when necessary, filtered (0.45 µm), and injected using BGE as a diluent. The standard, tablet, and stressed samples were analyzed under identical conditions. Degradation was assessed by comparing DAPA/IS peak area ratios to those of the standard quality reference (SQR), confirming method reliability and accuracy.

#### 2.4.2 Linearity and LOD and LOQ

Linearity was determined by constructing three standard curves, each with six concentrations of DAPA standard solution (50, 75, 100, 125, 150, and 175 µg mL^−1^), prepared in triplicate and with the IS in the fixed concentration of 75 µg mL^−1^ at all points. Linear regression was determined through analysis of variance (ANOVA). LOD and LOQ for DAPA were calculated using the standard deviation of the intercept (*σ*) and the slope (*S*) of the calibration curve: LOD = 3.3 (*σ*/*S*) and LOQ = 10 (*σ*/*S*).

#### 2.4.3 Precision

The precision of the method was established by repeatability (intraday) and intermediate precision (interday) studies. Both were determined by calculating the relative standard deviation (RSD) of the ratio between the areas of the DAPA sample solution (100 µg mL^−1^) and the areas of the IS (75 µg mL^−1^). The six DAPA sample solutions were prepared individually and injected in triplicate.

#### 2.4.4 Accuracy

Accuracy analysis was conducted by recovering known amounts of DAPA standard solution added to the sample solution, maintained at a constant concentration of 100 µg mL^−1^. The added amounts of DAPA standard were 25, 50, and 75 µg mL^−1^, in addition to IS at a 75 µg mL^−1^ concentration. Each solution was prepared in triplicate and injected three times. Recovery percentages were calculated using the following formula:

Recovery(%)=[(Cfound−Csample)/Cadded]×100

where *C*_found_ is the concentration measured in the spiked sample (sample plus added standard), *C*_sample_ is the concentration measured in the unspiked (original) sample, and *C*_added_ is the known concentration of the added standard.

#### 2.4.5 Robustness

During the initial optimization of the analytical method, several parameters were evaluated to confirm the method's suitability. Additionally, to verify the robustness of the analytical method, slight variations were performed, and the response results were compared with those of the normal condition. The parameters evaluated were capillary temperature (23–27°C), injection time (4–6 s), and applied voltage (13.5–14.5 kV).

### 2.5 Greenness Assessment

The Analytical Greenness Metric (AGREE) was used to assess the greenness profile of the developed CE method and to compare it with a reported HPLC method for determining DAPA. The pictograms were generated using the open‐source software available at https://mostwiedzy.pl/AGREE (version 0.5, 2020) [24].

## 3 Results and Discussion

### 3.1 Method Development

The initial method development focused on selecting an appropriate BGE to ensure reliable detection of the analyte in solution [25, 26, 27]. Several BGEs were evaluated—sodium phosphate monobasic, boric acid, TRIS–HCl, and sodium tetraborate—at concentrations of 10–50 mM and pH 4–11. Preliminary experiments revealed broad, low‐intensity, and asymmetrical peaks, particularly at non‐alkaline pH values. Only sodium tetraborate at 40 mM and pH 10.5 yielded sharp, intense peaks, making it the most suitable for further optimization. The selection of pH 10.5 was guided by the physicochemical properties of DAPA, which contains hydroxyl groups in the glycosidic moiety with a p*K*_a_ of approximately 12.57. According to the Henderson–Hasselbalch equation, the analyte remains predominantly in its neutral form at this pH, with only ∼0.85% deprotonated. The alkaline pH was therefore not intended to promote ionization but rather to enhance electroosmotic flow (EOF), which is essential for the migration of neutral or weakly ionizable species in CE. Moreover, under these conditions, the presence of tetraborate anions enables reversible complexation with glycosidic moieties in the analyte, modifying its hydrodynamic properties and improving migration behavior—a well‐documented phenomenon for glycosylated compounds [28]. Although the initial results were promising, the method produced high current (79.8 µA at 20 kV), leading to Joule heating, reduced reproducibility, and potential capillary degradation [29, 30]. To address this, adjustments were made to the applied voltage, BGE concentration, and capillary temperature to reduce current without compromising analytical performance. The final optimized conditions are summarized in Table 1.

**TABLE 1:** Electrophoretic conditions established for the determination of dapagliflozin (DAPA) in tablets.

Parameters	Description
Capillary	Fused silica capillary (48.5 cm total length, 40 cm effective length, 50 µm internal diameter)
Temperature	25°C
Preconditioning	3 min NaOH 0.1 M; 1.5 min H_2_O; 3 min electrolyte
Electrolyte	Sodium tetraborate 40 mM, pH 10.5
Injection	Hydrodynamic injection 50 mBar for 5 s
Applied voltage	14 kV
Current result	52 µA
Detection	230 nm

### 3.2 IS Selection

**FIGURE 2:**
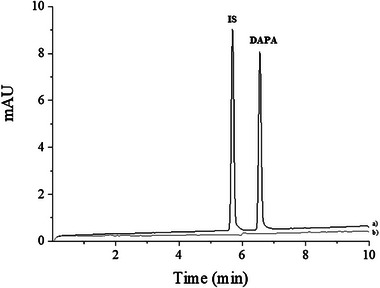
(a) Electropherogram obtained from injection of DAPA standard solution 75 µg mL^−1^) and internal standard (IS, 75 µg mL^−1^) and (b) excipient solution. Electrophoretic conditions: 48 cm fused silica capillary; 40 mM sodium tetraborate background electrolyte (BGE), pH 10.5; applied voltage of 14 kV; capillary temperature of 25°C and detection wavelength at 230 nm. DAPA, dapagliflozin.

After establishing the BGE composition that presented the best result for determining DAPA, the IS was selected. The purpose of using an IS is to compensate for injection errors and small fluctuations in the migration time of the analyte of interest. The use of IS in CE analyses is highly recommended to ensure the repeatability of the injections. Furthermore, they are also used to increase accuracy in identifying compounds through relative migration times [30]. The substances tested were ampicillin, atenolol, bupivacaine, lincomycin, chloroquine, and ranitidine. Figure 2 shows the electropherogram of DAPA with ranitidine as the IS, selected for its symmetrical, well‐resolved peak, consistent migration time, and absence of interference at 230 nm. The IS ensured reproducible DAPA/IS area ratios, validating its suitability for quantitative analysis. System suitability was evaluated by performing five consecutive injections of a standard solution containing 75 µg mL^−1^ of DAPA and IS. The method exhibited excellent performance, with high separation efficiency for DAPA (24 255 theoretical plates), satisfactory peak symmetry (1.01), and baseline resolution between analyte and IS (*R*_s_ = 5.4). The RSD was below 5%, confirming the precision and reliability of the analytical system. Migration times were approximately 5 min for the IS and 6.5 min for DAPA (Figure 2a).

### 3.3 Method Validation

#### 3.3.1 Selectivity

The analysis of the placebo solution showed no interference of the excipients present in the commercial product on the quantification of DAPA (Figure 2).

**FIGURE 3:**
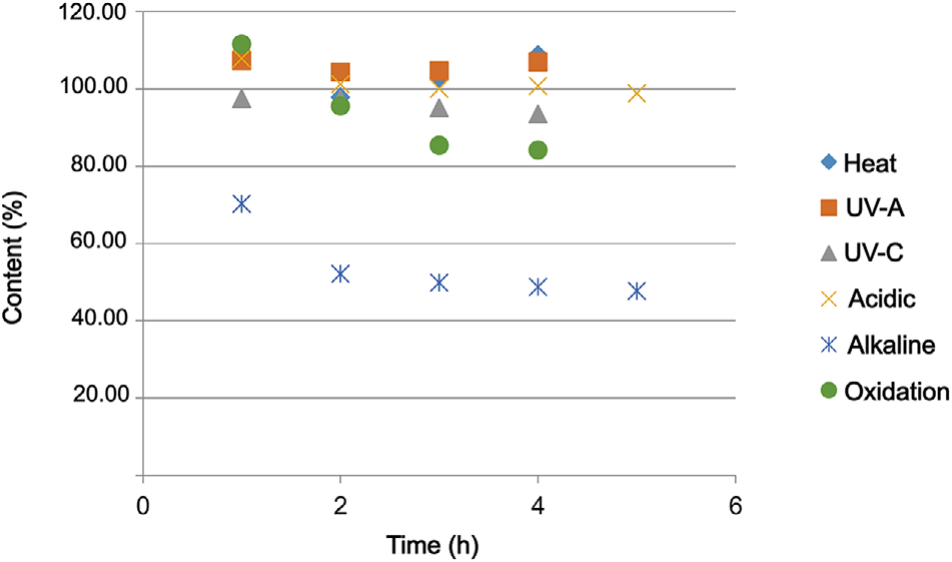
Graph representing the decay in DAPA content under different forced degradation conditions.

The visual analysis of the electropherograms in all stress conditions tested showed no formation of any additional degradation peaks. However, in quantification, a decrease in its content was noted when degraded in alkaline (close to 50%) and oxidative (around 20%) environments compared to the sample not degraded in the same concentration. On the other hand, the sample did not show a pronounced decrease in content when exposed to heat, radiation, and acidic environments. The following graph (Figure 3) illustrates the decay in drug concentration in each forced degradation condition. Peak purity was assessed using ChemStation software with DAD, which records UV–vis spectra throughout the elution. Spectral correlation coefficients near 1 confirmed peak homogeneity and absence of co‐elution, supporting the method's selectivity. Although no distinct degradation peaks were observed, this may reflect limited sensitivity to low‐level impurities. Nonetheless, validation confirmed satisfactory intra‐ and interday precision of the DAPA/IS ratio. A marked reduction in this ratio under stress conditions provided further evidence of analyte degradation.

#### 3.3.2 Linearity and LOD and LOQ

**FIGURE 4:**
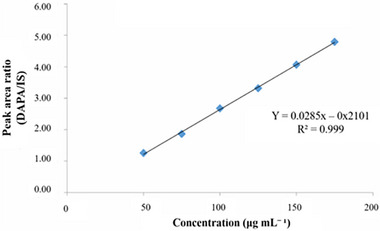
Calibration curve for DAPA obtained by CE in the concentration range of 50–175 µg mL^−1^. DAPA, dapagliflozin; IS, internal standard.

The regression equation of the standard curves was *y* = 0.0285*x* − 0.2101, *R* ^2^ equal to 0.999, demonstrating the linear relationship between DAPA concentrations and the responses generated (Figure 4). The analysis of the residues shows a normal distribution, being randomly distributed without atypical samples. Standardized residuals are also suitable as they are all between +2 and −2. The results of the ANOVA test for linear regression were considered satisfactory, and it can be stated that the proposed method has linearity in the studied concentration range (50–175 µg mL^−1^).

**FIGURE 5:**
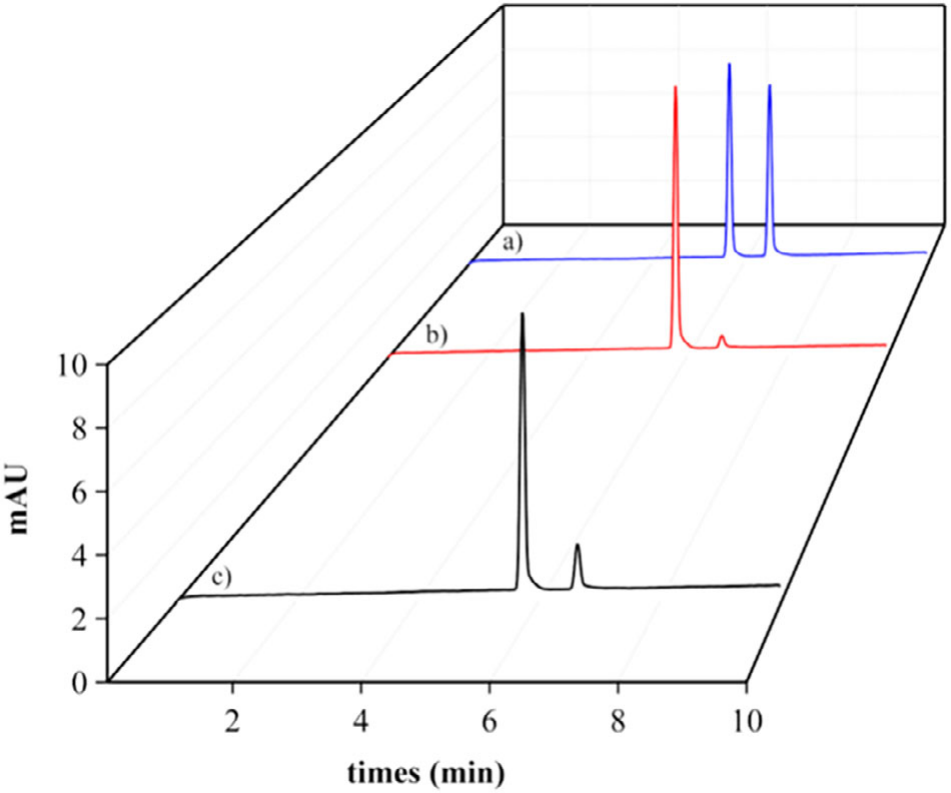
(a) Electropherogram of standard solution containing DAPA (75 µg mL^−1^) and IS (75 µg mL^−1^); (b) electropherogram at the LOD; and (c) electropherogram at the LOQ, as determined from the calibration curve.

The LOD and LOQ of the method, calculated from the standard curve data using the standard deviation of the intercept (*σ* = 0.0536) and the slope of the calibration curve, were 6.2 and 18.8 µg mL^−1^, respectively. The method was able to detect and quantify the analyte at the established LOD and LOQ levels with acceptable precision (RSD < 5%, *N* = 5), as confirmed by five replicate injections (Figure 5).

#### 3.3.3 Precision

The precision was confirmed to be over 18 injections, yielding an RSD of approximately 2.5%. Table 2 presents the results of the precision study, showing low variability both within the same day (repeatability, *n* = 6) and across different days (intermediate precision, *n* = 18). The RSD values obtained for intraday and interday analyses of DAPA were within acceptable limits, confirming the precision and reliability of the proposed CE method.

**TABLE 2:** Results obtained in repeatability (intraday) and intermediate precision (interday) tests.

Precision	Drug content (%)	RSD (%)
**Intraday (%)** ^a^		
Day 1	98.29	2.53
Day 2	97.00	2.06
Day 3	96.77	3.05
**Interdays (%)** ^a^	97.35	2.52

Abbreviation: RSD, relative standard deviation.

^a^ *n* = 18.

#### 3.3.4 Accuracy

The method demonstrated acceptable accuracy, with DAPA recovery averaging 102.72% across tested concentrations (125, 150, and 175 µg mL^−1^), all within the calibration range (Table 3). No signal saturation or non‐linearity was observed at the upper limit, confirming the method's reliability without requiring extension of the calibration range.

**TABLE 3:** Accuracy assay: Percentages of dapagliflozin (DAPA) recovery at three concentration levels.

Standard concentration added (µg mL^−1^)	Final concentration (µg mL^−1^)	Mean recovery (%)	RSD (%)
25	125	102.32	1.04
50	150	101.22	0.40
75	175	104.63	1.21

Abbreviation: RSD, relative standard deviation.

#### 3.3.5 Robustness

To assess robustness, small variations in temperature, injection time, and applied voltage were introduced. As shown in Table 4, these conditions are compared with the standard (unmodified) parameters. The relative area values (DAPA/IS) remained consistent across all conditions, indicating that such fluctuations did not significantly impact the method's performance. Additionally, parameters such as pH, BGE composition, and concentration, previously optimized due to their influence on EOF, further emphasize the importance of a robust and well‐controlled system.

**TABLE 4:** Experimental parameters adjusted in the robustness evaluation of the capillary electrophoresis (CE) method for dapagliflozin (DAPA) quantification.

Parameters	Average response (relative area)	Mean RSD (%)
Temperature—23°C	1.331	1.11
Temperature—27°C	1.336	1.55
Injection time—4 s	1.328	2.88
Injection time—6 s	1.378	1.93
Voltage—13.5 kV	1.399	0.64
Voltage—14.5 kV	1.406	0.26
Normal analysis conditions	1.399	0.64

Abbreviation: RSD, relative standard deviation.

### 3.4 Performance and Greenness Assessment of the CE Method Compared to HPLC

The proposed CE method was statistically compared with a previously reported HPLC method for DAPA quantification [31]. On the basis of repeatability data (*n* = 18) and an unpaired Student's *t*‐test, both methods showed comparable accuracy, with mean recoveries of 97.44% (CE) and 96.74% (HPLC), and no significant difference (*p* = 0.2463), confirming their analytical equivalence (Table 5). Although the CE method exhibited greater variance (5.71 vs. 0.55 for HPLC), this did not compromise its accuracy or precision, as reflected in the similar mean recoveries. Beyond analytical performance, the methods were compared in terms of operational and environmental aspects. Although HPLC offers faster analysis—approximately three times quicker—it requires higher solvent consumption and costly instrumentation and generates considerable waste [5, 6, 7, 8, 9, 10, 11, 12]. In contrast, CE, despite longer preparation and analysis times, is more economical and environmentally sustainable, using minimal reagent volumes and producing less waste. These characteristics align with GAC principles [17, 20, 24, 25, 26, 27], making CE a viable alternative in laboratories that prioritize sustainability and cost‐efficiency (Table 6) [31].

**TABLE 5:** Statistical comparison between high‐performance liquid chromatography (HPLC) and capillary electrophoresis (CE) methods for dapagliflozin (DAPA) determination, including calculated relative error [31].

	HPLC	CE
Mean	96.73943306	97.44333333
Variance	0.546476894	5.705647059
Observations	18	18
Hypothesized mean difference	0	
Degrees of freedom	20	
*t* Stat	1.194355446	
*p* (*T* ≤ *t*) one‐tail	0.123156049	
*t* Critical one‐tail	1.724718218	
*p* (*T *≤ *t*) two‐tail	0.246312098	
*t* Critical two‐tail	2.085963441	
Relative error (%)	—	0.73

**TABLE 6:** Comparison between high‐performance liquid chromatography (HPLC) and capillary electrophoresis (CE) methods in the quantification of dapagliflozin (DAPA) in tablets [31].

Parameter	HPLC	CE
Analysis time	4 min	8 + 7.5 min preconditioning
Preparation of the mobile phase/electrolyte	Mix acetonitrile and water; sonicate; filter	Sodium tetraborate weighing; dilution in water; pH adjustment; filtration
IS preparation	—	Standard weighing; dissolution in water
Sample preparation	Dissolve in acetonitrile; dilute in mobile phase	Dissolve in ACN; dilute in the electrolyte
Nature of solvents	Organic (70%) and aqueous (30%)	Aqueous
Expense of solvents per run	4 mL (2.8 mL of acetonitrile)	Insignificant (on the scale nanoliters)
Expense of solvents in method validation	0.5 L of acetonitrile	Insignificant (on the scale nanoliters)
LOD and LOQ	2.2 and 6.5 µg mL^−1^	6.2 and 18.8 µg mL^−1^

**FIGURE 6:**
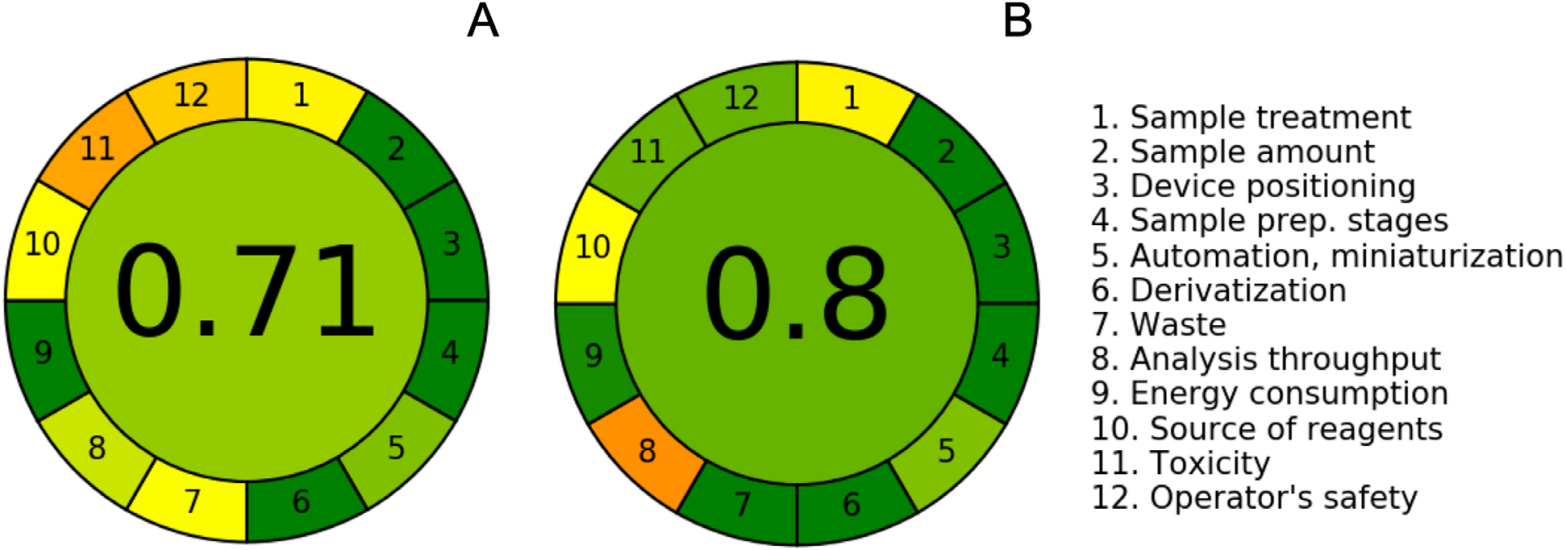
AGREE metric assessment results of (a) HPLC method and (b) CE method.

To further evaluate environmental impact, the AGREE metric was applied to both methods. This tool, based on the 12 principles of GAC, generates a colored pictogram with a score ranging from 0 to 1, reflecting overall greenness [3, 20, 24]. As shown in Figure 6, HPLC's lowest scores were associated with the use of acetonitrile and its associated waste (Principles 7 and 10–12), whereas CE's only notable drawback was its lower throughput (Principle 8). The CE method achieved a higher AGREE score (0.80) compared to HPLC (0.71), underscoring its superior environmental profile. Despite using a potentially hazardous solvent, the CE method employs it in minimal quantities, diluted within the BGE, which substantially reduces its environmental impact. This, combined with low waste generation and solvent consumption, highlights CE's alignment with green chemistry without compromising analytical performance.

## 4 Conclusion

The DAPA determination method developed by the EC demonstrated satisfactory performance for all analytical validation parameters, including specificity, linearity, precision, accuracy, robustness, and sensitivity limits. Compared to HPLC, CE offers greater sustainability and cost‐effectiveness due to reduced solvent use, energy consumption, and waste generation, as confirmed by green chemistry metrics. However, CE presents longer analysis times, lower throughput, and reduced sensitivity, which may limit its applicability in trace analysis. Despite these limitations, the method demonstrated accurate, precise, and selective quantification of DAPA, making it a suitable choice for routine pharmaceutical analysis, particularly in environments that prioritize sustainability and operational economy.

## Conflicts of Interest

The authors declare no conflicts of interest.

## Data Availability

All data underlying the findings of this study are available from the corresponding author upon reasonable request.
